# Phoenics: a novel statistical approach for longitudinal metabolomic pathway analysis

**DOI:** 10.1186/s12859-025-06118-z

**Published:** 2025-04-16

**Authors:** Camille Guilmineau, Marie Tremblay-Franco, Nathalie Vialaneix, Rémi Servien

**Affiliations:** 1 INRAE, University of Montpellier, LBE, 102 Avenue des Etangs, 11100 Narbonne, France; 2 INRAE, Université de Toulouse, ENVT, Toxalim, 31027 Toulouse, France; 3 Axiom Platform, MetaToul-MetaboHUB, National Infrastructure for Metabolomics and Fluxomics, 31027 Toulouse, France; 4 Université de Toulouse, INRAE, UR MIAT, 31326 Castanet-Tolosan, France

**Keywords:** Mixed model, Longitudinal data, Metabolomics, Metabolic pathways

## Abstract

**Background:**

Metabolomics describes the metabolic profile of an organism at a given time by the concentrations of its constituent metabolites. When studied over time, metabolite concentrations can help understand the dynamical evolution of a biological process. However, metabolites are involved into sequences of chemical reactions, called metabolic pathways, related to a given biological function. Accounting for these pathways into statistical methods for metabolomic data is thus a relevant way to directly express results in terms of biological functions and to increase their interpretability.

**Methods:**

We propose a new method, phoenics, to perform differential analysis for longitudinal metabolomic data at the pathway level. In short, phoenics proceeds in two steps: First, the matrix of metabolite quantifications is transformed by a dimension reduction approach accounting for pathway information. Then, a mixed linear model is fitted on the transformed data.

**Results:**

This method was applied to semi-synthetic NMR data and two real NMR datasets assessing the effects of antibiotics and irritable bowel syndrome on feces. Results showed that phoenics properly controls the Type I error rate and has a better ability to detect differential metabolic pathways and to extract new impacted biological functions than alternative methods. The method is implemented in the R package phoenics available on CRAN

## Background

Metabolomic datasets provide the amount of small molecules, called metabolites, that are present in complex mixtures at a given time. Metabolomics gives access to functional information due to its proximity to the phenotype [1, 2]. It is also a non-invasive method when performed on easily accessible biological samples, such as urine or blood [3]. Metabolomic analyses are used in various areas, such as biomarker discovery in precision medicine [4] or cancer diagnosis [5].

It is thus not surprising that more and more experiments target the evolution of the metabolome over time, in different conditions, or following an event of interest [6–8]. In these cases, metabolomic data are acquired at several time points on the same individuals. Here, we target such cases and we address the question of using time-course metabolomic data acquired in several experimental conditions to extract biomarkers of either the differences between conditions or of the time effect. This question is illustrated on a typical use case in Sect. “Analysis of antibiotics effect with phoenics”, where biomarkers of the effect of an antibiotic treatment over time are obtained from feces metabolome.

A common approach to analyze longitudinal multivariate data is to rely on mixed linear models [9]. This type of model is well adapted to longitudinal data because it can incorporate a random individual effect, accounting for the dependency between repeated measurements on the same individuals. The fixed effects thus correspond to controlled effects and effects of interest, as experimental conditions or time in longitudinal analyses. In addition, in metabolomics, this type of model is frequently combined with dimension-reduction techniques, due to the high dimensionality of the data [10].

However, metabolites are functionally grouped into sequences of chemical reactions, called metabolic pathways. Accounting for pathways into statistical methods for metabolomic data is thus a good way to directly express results in terms of biological functions and to increase their interpretability. Typically, this is done by enrichment analysis (or Over-Representation Analysis (ORA)), which consists of performing tests independently for each metabolite and combining the results of these tests to extract pathways containing more differential metabolites than what would have been expected by chance [11]. A drawback is that this approach does not account for the correlation between metabolites within a pathway, which can lead to a loss of information about the overall pathway dynamics.

In addition to enrichment analysis, Functional Class Scoring (FCS) methods have been used to perform differential analysis. Most of these methods have been developed in the field of transcriptomics, to perform tests at a gene set level, but they can be easily extended to metabolomic pathway analysis. Maleki et al. [12] propose an exhaustive review of these approaches in the field of transcriptomics. They identify a large number of univariate FCS methods, e.g., Mootha et al. [13–18], in which a gene score is typically computed for each gene and then gene scores are used to calculate a gene set score and to derive a *p*-value. However, apart from the method of Jiang and Gentleman [18], these approaches are only adapted to compare two conditions and can not handle longitudinal data and repeated measurements. In addition, even if the method of Jiang and Gentleman [18] can be adapted to more complex settings and can also adjust the results for fixed effect covariates, it is not designed to handle random effects and repeated measurements.

In contrast to univariate FCS methods, multivariate FCS methods directly calculate gene set scores from the original data. Calculating a gene set score instead of gene scores allows leveraging the overall pathway dynamics and thus increases the sensitivity in pathway detection, especially when the signal in the original data is low [19]. Indeed, approaches like enrichment analysis are based on the power of a primary analysis where metabolites are analyzed independently and are therefore unable to detect differential pathways that correspond to an accumulation of small effects on metabolites (found non-differential due to a lack of power, for instance). Furthermore, a gene set score approach reduces the dimensionality of the data, as the number of gene sets is smaller than the number of genes. In the field of metabolomics, Wieder et al. [19, 20] develop one of these methods, where they transform the data at the metabolite level to data at the pathway level before performing differential analysis on this transformation. In the field of transcriptomics, multivariate FCS methods include the work of Goeman et al. [21, 22] based on multivariate GLM or on Hotelling’s test [23] but again, this proposal can not account for covariates. More recently, Ozier-Lafontaine et al. [24] proposes a nonlinear test for gene sets based on a kernel approach. In its current available implementation, the test is also restricted to test differences between two conditions without covariates.

Here, we present an extension of the method of Wieder et al. [20] to allow for the analysis of longitudinal repeated measurements. Our method, called phoenics proceeds in two steps: a data transformation similar to the one of Wieder et al. [20] and a mixed model. The method is benchmarked against enrichment analysis (ORA), ktest [24], and globaltest [21] on semi-synthetic and experimental NMR data in Sect. “Results and discussion”. The method is implemented in the R package phoenics available on CRAN.^1^ The package allows to use any kind of pathway information provided by the user. In the current implementation of the package, an automatic research of the pathways can be perfomed in the KEGG database.

## Methods

In the following, *X* is a $(nT \times m)$-matrix of quantifications, where *n* is the number of individuals, *T* the number of time points (hence, the total number of observations is equal to *nT*), and *m* the number of metabolites. *X* is organized such that its *n* first rows correspond to the quantification measurements of the *n* individuals at the first time point. In addition, individuals can belong to different conditions of interest or to different groups of controlled conditions that form, along with time, the fixed effects. Here, we target the question of the test of a given fixed effect accounting for repeated measurements and pathway information on the metabolites.

In general, such questions are handled by performing independent tests of the targeted fixed effect with linear mixed models and then by post-processing the results of these tests with an enrichment analysis [16]. Enrichment analysis consists of splitting metabolites into significant and non-significant groups according to the results of the individual tests and in computing a cross table between this information and the inclusion in a given pathway, $\mathcal {M}_l$. The pathway *p*-value is finally obtained with a Fisher exact test on this cross table. A “background” metabolite set is usually used to define the set of all achievable metabolites (e.g., the quantified metabolites) and its definition is known to have a strong impact on the results of enrichment analysis. In particular, using a non-specific background (all known metabolites of a given organism) can lead to a large number of false positives [25].

### Description of the proposed approach

#### Phoenics method

Each metabolite belongs to one or several metabolic pathways $\mathcal {M}_l$. The proposed method is made of two steps:

1. *Factor analysis representation to extract pathway signatures* For each metabolic pathway $\mathcal {M}_l$, and similarly to Wieder et al. [20], a PCA is performed on the metabolite quantification matrix of this pathway, $Z_l = (X_{ij})_{i = 1,\ldots ,nT,\, j \in \mathcal {M}_l}$. The scores of the $m^{*}_{l}$ first principal components are extracted in the $(nT \times m_l^*$)-matrix $A_l$. In the following, to simplify the notations, we will generically refer to *a* as one of the columns of one of the matrix $A_l$, which corresponds to one of the principal components for a given pathway.
2. *Mixed model to test pathways* For each principal component *a*, the following mixed model is then estimated:
  1 $$\begin{aligned} a = \beta _t + \theta _d + u_i + \epsilon _{tdi} \end{aligned}$$
  with
  $\beta _t$ the fixed effect of time *t*;
  $\theta _d$ the fixed effect of condition *d*;
  $u_i\sim \mathcal {N}(0, G)$ the random effect of individual *i*, with *G* the covariance matrix;
  $\epsilon _{tdi} \sim \mathcal {N}(0, \sigma _{\epsilon }^{2})$ the residuals of the model, which are assumed to be i.i.d.

The significance of a given fixed effect *f* is then tested using the likelihood ratio test comparing the full model of Equation (1) to a restricted model that does not contain the effect *f*. To test the time effect, the restricted model is

$$a = \theta _d + u_i + \epsilon _{tdi},$$

and to test the condition effect, the restricted model is

$$a = \beta _t + u_i + \epsilon _{tdi}.$$

For a given metabolic pathway $\mathcal {M}_l$, this estimation is repeated for each column of $A_l$, which results in $m^{*}_{l}$ *p*-values. The Simes’ procedure [26] is used to aggregate the $m^{*}_{l}$ *p*-values: This procedure controls the Type I error of the null hypothesis H$_0 = \cap _{j=1}^{m^*_l}$ H$_{0j}$, where H$_{0j}$ is the absence of effect of *f* on the *j*th column of $A_l$.

Note that the Simes’ procedure relies on the assumption of independence or Positive Regression Dependence on a Subset (PRDS) among tests, which is known to be difficult to assess in practical situations. However, the procedure is known to be robust to deviations from independence [27, 28] and PRDS is also the weakest known assumption under which the False Discovery Rate (FDR) is controlled by the Benjamini and Hochberg procedure [29, 30]. Hence, despite the fact that there is no formal guarantee that principal components satisfy the PRDS property, we consider the use of the Simes’ procedure reasonable due the robust nature of the PRDS condition.

As a consequence, this approach leads us to obtain a single *p*-value by metabolic pathway for each tested effect *f*. Finally, since multiple *p*-values are obtained (one for each pathway), an additional multiple test correction is required. We chose to control the FDR by using the Benjamini-Hochberg (BH) procedure [29].

In practice, the method only requires to choose the parameter $m_l^*$, corresponding to the number of principal components on which mixed models are estimated for each pathway. To allow the method to retain enough information in the data to estimate each possible effect, we propose to set $m_l^*$ equal to the number of fixed effects, *F*. This choice assumes that all technical bias effects have been removed prior to the data analysis.

#### A variant based on partial PCA

A limitation of the approach described above is that PCA does not account for the specific structure of the data where some observations (typically those corresponding to measurements of a given individual) might be more correlated than others. Hence, to account for this particular structure of the correlation dependency between data, we also propose to replace the global PCA by independent PCA analyses, in the same spirit than the multi-table analysis called Multiple Factor Analysis (MFA) [31].

In this version, the metabolite quantification matrix of the tested pathway is viewed as a set of matrices, each corresponding to a given time point, *t*: $Z_l^{(t)} = (X_{ij})_{i = n(t-1)+1,\ldots ,nt,\, j \in \mathcal {M}_l}$ for $t = 1, \ldots , T$. MFA then performs an individual (called partial) PCA of each matrix and eventually average them, weighting each PCA by its first eigenvalue so as to balance each block’s contribution.

Here, we stick to the first step of the MFA and retrieve the scores of the $m^{*}_{l}$ first principal components of the partial analyses to stack them into a $(nT \times m_l^*$)-matrix $A_l$. The second step (corresponding to the mixed model) is then performed in the same way as in Sect. “Phoenics method”.

Note, however, that while this approach is better appropriate to test the condition effect, it is not relevant for the time effect, as the latter is expected to be erased by performing the independent partial analyses.

### Experimental and semi-synthetic datasets

#### Effect of antibiotics in mice

The method performances were assessed using experimental data from Choo et al. [32], available in the MetaboLights metabolomics data repository [33] with the identifier MTBLS422. This study investigates the changes in fecal metabolome induced by antibiotics. The data contains nuclear magnetic resonance (NMR) spectra obtained from $2 \times 8$ mouse feces-based fluid subjected or not to an antibiotics treatment (vancomycin-imipenem). In the sequel, the treatment status of the mouse is called the “condition” and is a fixed effect of interest (e.g., metabolite quantification differences between conditions are tested). In addition, in each condition, spectra have been acquired from samples collected at three time points (5.5, 7.5, or 9), and the time fixed effect thus corresponds to a second fixed effect of interest. The dataset contains 46 observations (the design is not complete: 2 observations are missing compared to the complete design).

Metabolite quantifications were estimated from 1D $^1$H NMR spectra with the R package **ASICS** [34]. This resulted in the quantification of 176 metabolites in total. These quantifications represent the relative abundance of metabolites within samples. Metabolite pathways were retrieved using the R package **KEGGREST** [35], which queries the KEGG database [36] for pathways specific to a given organism (here, *Mus musculus*, with KEGG Pathway database from release 109), as implemented in our package **phoenics**. A total number of 98 pathways were obtained, containing at least two quantified metabolites for a total of 128 metabolites found in the pathways. The average number of metabolites in the different pathways is just above 6 (with a maximum of 32) whereas each metabolite is included in average in more than 4 pathways, with a maximum of 31. This highlights the fact that, most pathways share some common metabolites, i.e., they overlap.

#### Effect of irritable bowel syndrome in human

A second experimental dataset, from Mars et al. [37], was used to assess the method performances. Data are available in the MetaboLights metabolomics data repository [33] with the identifier MTBLS1396. This study conducts a longitudinal analysis to investigate the gut metabolome in the context of irritable bowel syndrome. The 1D $^1$H NMR spectra were obtained from $51 \times 2$ human stool samples affected or not by the irritable bowel syndrome (IBS-C). In each condition, samples have been collected at 8 time points. The condition and the time effects correspond to the fixed effects of interest.

The R package **ASICS** [34] was used to estimate metabolite quantifications, resulting in 169 metabolites. Using the R package **KEGGREST** [35], a total of 104 metabolic pathways were retrived from KEGG database [36] (*Homo sapiens* specific database, with KEGG Pathway database from release 113), containing at least two quantified metabolites for a total of 124 metabolites found in the pathways.

#### Performances assessment through simulations

In addition to their analysis with **phoenics**, mice data were used to generate several semi-synthetic datasets to assess three criteria:

- *The control of Type I error rate.* Semi-synthetic datasets were simulated under the null hypothesis by erasing the effect of interest. These datasets are named **SimulatedH0**.
- *The statistical power.* A targeted signal was added in some pathways. More precisely, $k = 3$ pathways were randomly selected, in which a difference for the effect of interest was introduced. Several semi-synthetic datasets, referred as **SimulatedH1**, were generated according to different scenarios corresponding to different effect sizes. In order to assess the method variability, the random selection of the *k* metabolic pathways was repeated 100 times for each scenario.
- *The statistical power with respect to the percentage of differential metabolites.* The largest pathway in the semi-synthetic dataset was utilized to assess the percentage of differential metabolites required within a pathway for it to be detected as significant. This pathway, named “ABC transporters”, consists of 32 metabolites. Similarly to **SimulatedH1**, a difference between the levels of the effect of interest was introduced for $\tilde{p}$ selected metabolites in “ABC transporters” pathway (for $\tilde{p}$ varying from 1 to the maximum possible number, 32). To obtain results comparable with the previous simulations, a difference was also introduced for all of the metabolites in $k - 1$ other (randomly selected) pathways, using the same simulation process. These datasets are further referred to as SimulatedVSize. The choice of the $\tilde{p}$ differential metabolites in “ABC transporters” and of the other $k-1$ pathways was randomly replicated 20 times to also assess the variability of the results.

Datasets simulating separately the time and the condition as the effect of interest were first generated to test each effect individually. Then, a third dataset was used to test both time and condition effects simultaneously, where differences for both effects were introduced. The details of the simulation processes, depending on the effect of interest, are provided in the following paragraphs.

#### Testing the condition effect

The simulation process under the null hypothesis of this dataset, named SimulatedH0_Condition, is based on an adaptation of the method described in Wieder et al. [20]: the condition effect was erased by randomly permuting condition status among samples. The time status was not permuted to preserve the within-individual correlation structure. Thus, the time effect from the original dataset (if present) remain in the simulated data.

To simulate SimulatedH1_Condition and SimulatedVSize_Condition, the difference between conditions was artificially introduced in the selected metabolites, by simulating new quantifications as:

2 $$\begin{aligned} \forall \, i=1,\ldots ,nT,\ \forall \, d=1,\ldots ,D,\qquad \tilde{X}_{ij} = X_{ij} \times \gamma _d \end{aligned}$$

with $j \in \cup _{l=1}^k \mathcal {M}_l$, $X_{ij}$ the quantification of metabolite *j* for individual *i* at time *t* and $\gamma _d$ a chosen factor controlling the effect size in condition *d*. Thus, in this simulation, the artificial condition effect is expected to be detected in the *k* pathways while a time effect may also be present. Several scenarios were simulated, corresponding to different $\gamma _d$. The values chosen for $\gamma _d$ are provided in Table 1.

**Table 1:** SimulatedH1_Condition values of $\gamma _d$ according to the value of *d* and to the scenario

	Scenario 1	Scenario 2	Scenario 3
$d =$ “control”	1	1	1
$d =$ “vancomycin-imipenem”	10	3	2

#### Testing the time effect

In simulation SimulatedH0_Time, the time effect was erased by replacing the metabolite quantifications at time points 7.5 and 9 by the metabolite quantifications at time point 5.5. Then, a noise $b_{ij}$ was added to the metabolite quantifications at all time points, with $b_{ij} \sim \mathcal {N}(0, \sigma _{b}^{2})$, $\sigma _{b}^{2} = 0.05 \times [max(X) - min(X)]$ where *X* is the metabolite quantification matrix.

For simulations SimulatedH1_Time and SimulatedVSize_Time, the introduction of an artificial condition effect to chosen pathways is performed by simulating the quantifications as:

3 $$\begin{aligned} \forall \, i=1,\ldots ,nT,\ \forall \, t=1,\ldots ,T,\qquad \tilde{X}_{ij} = S_{ij} \times \gamma _t + b_{ij} \end{aligned}$$

where

- $$S = \begin{bmatrix} (X_{ij})_{i \in [1, \ldots , n]} \\ \vdots \\ (X_{ij})_{i \in [1, \ldots , n]} \end{bmatrix}$$ is a $(nT \times m)$-matrix where the $(X_{ij})_{i \in [1, \ldots , n]}$ matrix, which corresponds to the quantification of each metabolite at the first time $t = 5.5$, is repeated *T* times;
- $$j \in \cup _{l=1}^k \mathcal {M}_l$$;
- $\gamma _t$ is a chosen factor controlling the effect size at time step *t*;
- $$b_{ij} \sim \mathcal {N}(0, \sigma _{b}^{2})$$ where $$\sigma _{b}^{2} = 0.05 \times [max\big (S_{ij} \times \gamma _t\big ) - min\big (S_{ij} \times \gamma _t\big )]$$.

The values chosen for $\gamma _t$, corresponding to the different scenarios, are provided in Table 2.

**Table 2:** SimulatedH1_TimeValues of $\gamma _t$ according to the value of *t* and to the scenario

	Scenario 1	Scenario 2	Scenario 3	Scenario 4
$t =$ 5.5	1	1	1	1
$t =$ 7.5	5	2	1.5	1.2
$t =$ 9	10	3	2	1.5

#### Testing the condition and the time effects simultaneously

To simulate SimulatedH0_ConditionTime, the condition effect was first erased in the same way than in simulation SimulatedH0_Condition. Then, to erase the time effect, the same process than in simulation SimulatedH0_Time is used.

Then, the introduction of artificial effects for SimulatedH1_ConditionTime and SimulatedVSize_ConditionTime also combines the two previous simulation processes by first simulating the time effect as in Equation (3) and then adding a condition effect using Equation (2).

### Evaluation methodology

#### Comparison with existing methods

We compared phoenics to the reference method for metabolic pathway analysis, which is enrichment analysis (also called ORA) [16, 38]. The background set was chosen as the set of all identifiable metabolites, e.g., all metabolites in the reference database of the package ASICS (180 metabolites). The significance of metabolites was tested using the same linear mixed model as in phoenics. Metabolite *p*-values were corrected for multiple testing using BH correction, except for H$_0$ scenarios where raw *p*-values were used (since no signal is expected, BH correction at metabolite level is expected to filter out all metabolites). Fisher’s exact test was then used to calculate the pathway *p*-values and BH correction was used to account for multiple testing across pathways.

phoenics was also compared to a multivariate FCS method, **ktest** [24]. This method relies on kernel-based testing. More precisely, it compares the distribution of gene sets expression transformed by a kernel Fisher Discriminant Analysis (kFDA) [39] using a Hotelling’s test [40] in the kernel feature space. However, this approach is restricted to two conditions only. Therefore, for the test of time effect, pairwise comparisons of the three dates were conducted using the first two dimensions. The first two dimensions were also used for the test of condition effect. Similarly to what is done in phoenics, the Simes procedure was used to aggregate the six (2 dimensions $\times \, 3$ pairs of dates) or two (2 dimensions $\times \, 1$ pair of conditions) resulting *p*-values. Again, BH correction was used afterwards to account for multiple testing across pathways.

We also compared phoenics to another multivariate FCS method, globaltest [21]. This method relies on generalized linear model [41] and allows to test the association between groups of genes and an outcome, while accounting for covariates. The resulting *p*-values were corrected for multiple testing across pathways using the BH method.

#### Assessing method quality from simulated datasets

The control of Type I error was assessed using SimulatedH0 datasets. The percentage of pathways detected as positive by phoenics was calculated.

Metabolic pathways generated in SimulatedH1 have been classified into categories, presented in Table 3, according to whether they have been explicitly simulated as differential for the targeted effect or not. However, due to the overlap among pathways, our simulation of differential pathways resulted in a (small or large) proportion of metabolites in other pathways also being differential. Then, a third category has been defined for these overlapping pathways to which we can not give a clear status (differential or not). The number of simulated pathways in each category is presented in Table 3.

**Table 3:** SimulatedH1. Number and categories of simulated pathways

	Significant	Not significant	Number of simulated pathways (Condition)	Number of simulated pathways (Time)	Number of simulated pathways (ConditionTime)
Differential	True positive	False negative	300	300	300
Not differential	False positive	True negative	3175	3071	3055
Overlapping pathways	Positive	Negative	6325	6429	6445

The method quality was assessed by counting the number of pathways in each category of Table 3, among the 100 generated datasets. Precision (or positive predictive value, PPV), corresponding to the number of true positives over the total number of pathways declared positive, and recall (or sensitivity), corresponding to the number of true positives over the number of true differential pathways, were also calculated.

Finally, using SimulatedVSize, the percentage of significant “ABC transporters” pathways across the repetitions was computed and compared to the number of metabolites simulated as differential in this pathway.

The performances of the different methods on the overlapping pathways are difficult to analyze as we have no ground truth for them. To provide some results on these pathways, we compared the distributions of the percentage of differential (and non-differential) metabolites in the overlapping pathways. A relevant method should be able to differentiate overlapping pathways with a high number of metabolites simulated as differential (expected to be detected as differential) from overlapping pathways with a small number of metabolites simulated as differential (expected to not be detected as differential).

All the scripts used to perform the analyses of this article are available at https://forgemia.inra.fr/panoramics/rlib_bmcbioinformatics_2024.

## Results and discussion

In the following, phoenicsPCA will refer to the PCA-based approach, while the MFA based approach will be referred to as phoenicsMFA.

### Testing the condition effect

#### Assessing the control of Type I error

Table 4 gives the number of tests (falsely) detected under the null hypothesis (at a significance level of 5%) on the dataset **SimulatedH0_Condition**. It demonstrates that the Type I error is controlled by all the methods when testing the condition effect.

**Table 4:** SimulatedH0_Condition. Percentage of pathways detected as (or tests declared) positive under the null hypothesis simulation setting for a *p*-value threshold of 5%

	Condition
phoenicsMFA	1.10%
phoenicsPCA	4.08%
Enrichment	2.04%
**ktest**	0.00%
**globaltest**	0.00%

#### Assessing the performances of phoenics compared to alternative approaches

**Fig. 1:**
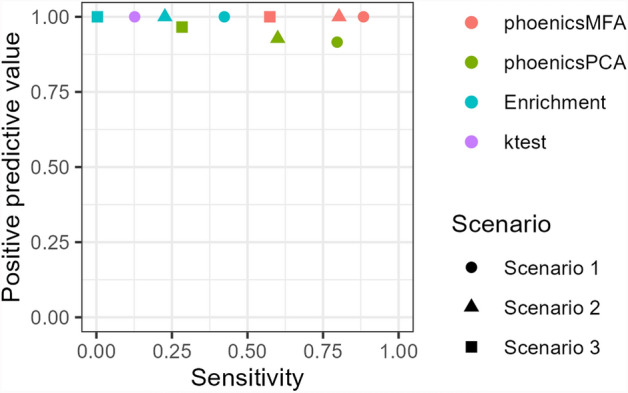
SimulatedH1_Condition. Positive predictive value (PPV) and sensitivity. Globaltest does not appear in the figure since it detects no positive pathways therefore no PPV can be calculated

Table 5 presents the number of pathways declared positive for the condition effect by phoenicsPCA, phoenicsMFA, enrichment analysis, ktest, and globaltest in each pathway category for all three scenarios (SimulatedH1_Condition). These results are further illustrated in Fig. 1, comparing the PPV and sensitivity of the five methods. Across scenarios, the sensitivity is higher with phoenicsMFA and phoenicsPCA than with enrichment analysis, ktest, and globaltest since phoenicsMFA and phoenicsPCA consistently detect a larger number of true positive pathways compared to the enrichment analysis and ktest. Additionally, as the simulated effect size decreases from Scenarios 1 to 3, the number of true positive pathways detected also decreases. phoenicsPCA and phoenicsMFA yield similar results in Scenario 1, but the difference between them increases in Scenarios 2 and 3. More specifically, in Scenario 3, phoenicsMFA identifies 54% of the differential pathways as positive, whereas only 28% of these pathways are identified as positive by phoenicsPCA. Furthermore, phoenicsMFA, the enrichment analysis, ktest, and globaltest exhibit no false positives (their PPV is equal to 1), contrary to phoenicsPCA (that thus has a lower PPV). Overall, these findings highlight the larger power of phoenicsMFA compared to the other methods for detecting differential pathways, especially in cases where the signal in the data is weaker, and without compromising the correct control of the Type I error.

phoenicsMFA and phoenicsPCA declare positive, respectively, 50.8% and 43.6% of the overlapping pathways in Scenario 1, while only 9.2% and 3.1% are positive with enrichment analysis and **ktest**, respectively. The percentage of positive overlapping pathways decreases in Scenarios 2 and 3 but phoenicsMFA and phoenicsPCA consistently identify the highest percentage of positive overlapping pathways.

**Table 5:** SimulatedH1_Condition. Counts of pathways in each category with semi-synthetic data simulation Scenario 1, 2, and 3. The percentages of pathways corresponding to the counts can be found in Additional File 1: Table S1

		True positive	True negative	False positive	False negative	Positive (overlap)	Negative (overlap)
Scenario 1	phoenicsMFA	251	2789	0	33	3065	2962
	phoenicsPCA	239	3153	22	61	2762	3563
	Enrichment	127	3175	0	173	584	5741
	**ktest**	38	3175	0	262	199	6126
	**globaltest**	0	3175	0	300	0	6325
Scenario 2	phoenicsMFA	228	2789	0	56	2327	3700
	phoenicsPCA	180	3161	14	120	1886	4439
	Enrichment	68	3175	0	232	353	5972
	**ktest**	0	3175	0	300	0	6325
	**globaltest**	0	3175	0	300	0	6325
Scenario 3	phoenicsMFA	163	2789	0	121	1631	4396
	phoenicsPCA	85	3172	3	215	1018	5307
	Enrichment	1	3175	0	299	2	6323
	**ktest**	0	3175	0	300	0	6325
	**globaltest**	0	3175	0	300	0	6325

**Fig. 2:**
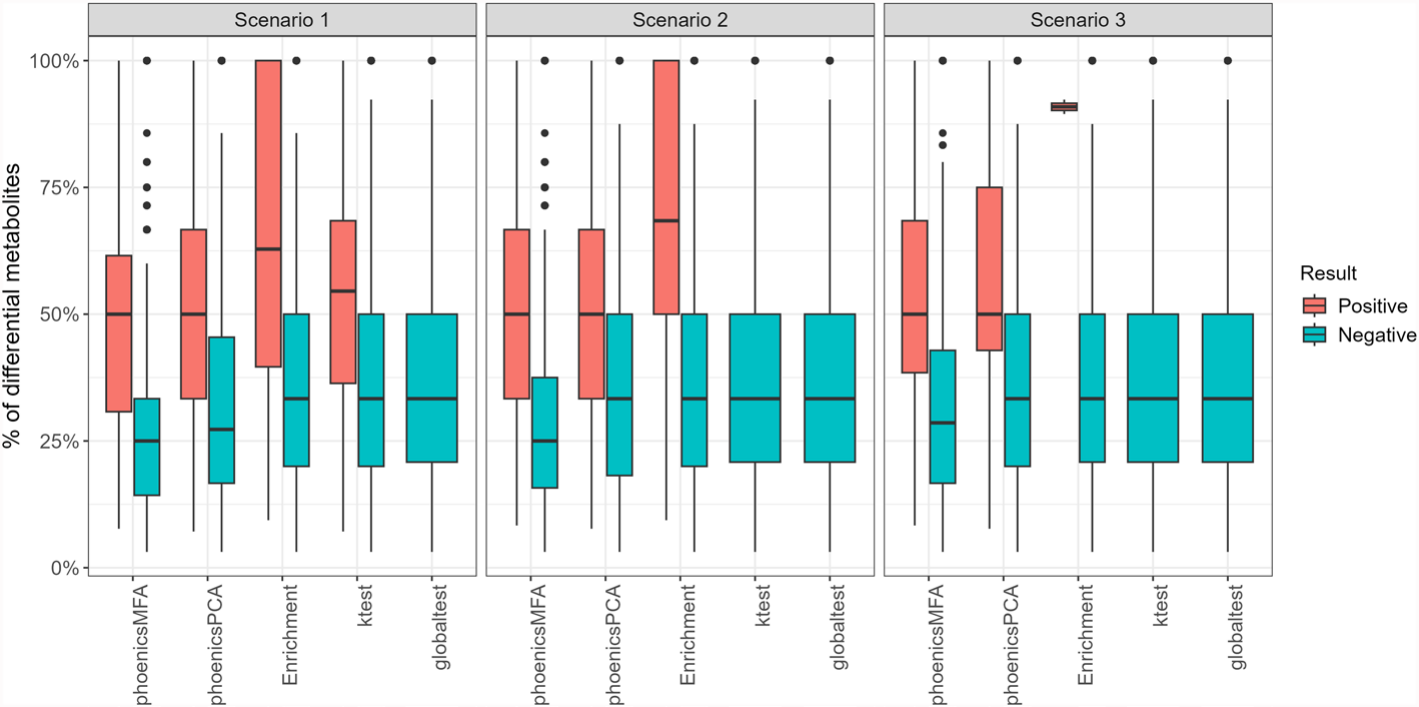
SimulatedH1_Condition. Percentage of differential metabolites in the overlapping pathways. Some of these pathways have a percentage of differential metabolites equal to 100% because they are included in a differential pathway

To further investigate the relevance of positive overlapping pathways, Fig. 2 illustrates the percentage of differential metabolites in the overlapping pathways, categorized as positive or negative by the five methods and three scenarios. As expected, the percentage of differential metabolites is higher in the positive overlapping pathways compared to negative pathways across all methods and scenarios. The positive overlapping pathways identified by phoenicsMFA have a lower percentage of differential metabolites compared to those identified by phoenicsPCA, enrichment analysis, and **ktest**. In addition, the positive overlapping pathways identified by phoenicsMFA show a percentage of differential metabolites that is more distinct from that of the negative overlapping pathways, compared to the other methods. This is consistent with the larger detection power of phoenicsMFA.

#### Assessing the detection power

**Fig. 3:**
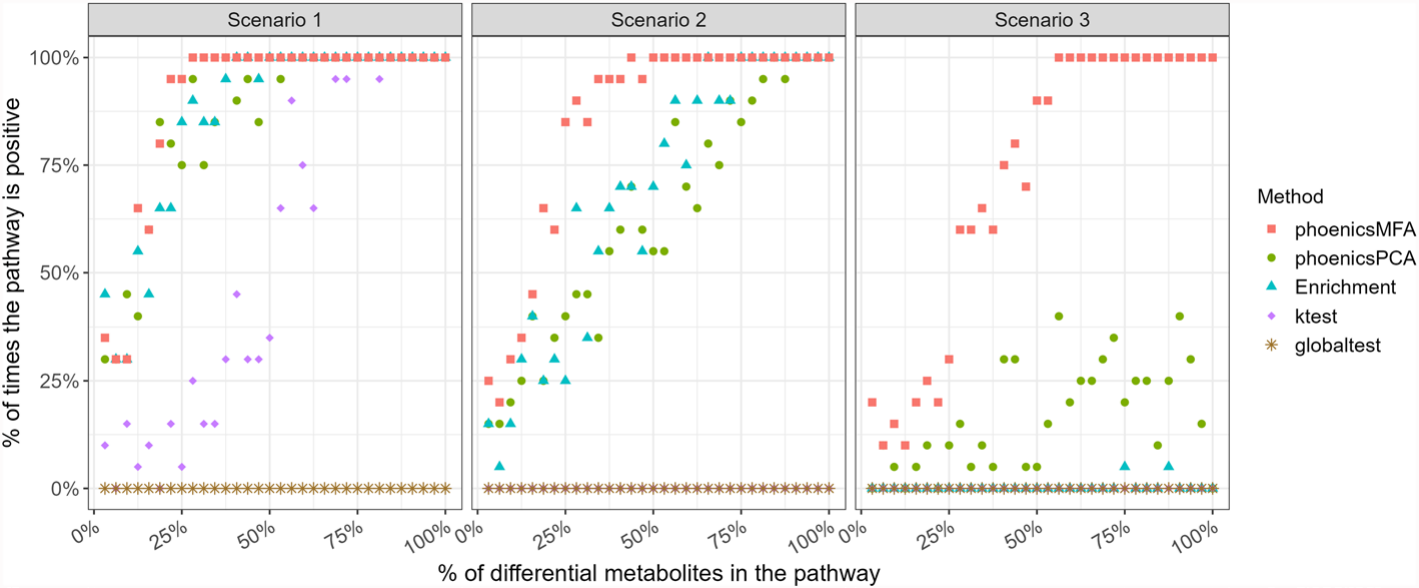
SimulatedVSize_Condition. Percentage of times the pathway “ABC transporters” is detected positive over the 20 simulations with respect to the percentage of differential metabolites in the pathway

Finally, Fig. 3 shows the detection power of the five methods with respect to the percentage of metabolites detected as differential in the pathway (**SimulatedVSize_Condition**). Again, phoenicsMFA shows a higher percentage of detection for a given percentage of metabolites simulated in the pathway. In addition, the difference between phoenicsMFA and the other methods increases as the effect size decreases (from Scenarios 1 to 3). Also, as expected, when the effect size is low (Scenario 3), a larger percentage of differential metabolites is required for the pathway to be declared significant. These results are consistent with the previous ones and confirm that phoenicsMFA has a larger detection power, especially when the effect size is low. Therefore, phoenicsMFA is well-suited to test the condition effect.

### Testing the time effect

The number of tests (falsely) detected under the null hypothesis (at a significance level of 5%) on the dataset **SimulatedH0_Time** demonstrates that the Type I error is controlled by all methods but **globaltest** (slightly above the 5% threshold), when testing the time effect (Table 6). In addition, the Type I error with phoenicsPCA is close to the 5% threshold, suggesting that phoenicsPCA has a better power than the other methods of analysis.

**Table 6:** SimulatedH0_Time. Percentage of pathways detected as (or tests declared) positive under the null hypothesis simulation setting for a *p*-value threshold of 5%

	Time
phoenicsMFA	0.00%
phoenicsPCA	4.08%
Enrichment	1.02%
**ktest**	0.00%
**globaltest**	5.10%

**Fig. 4:**
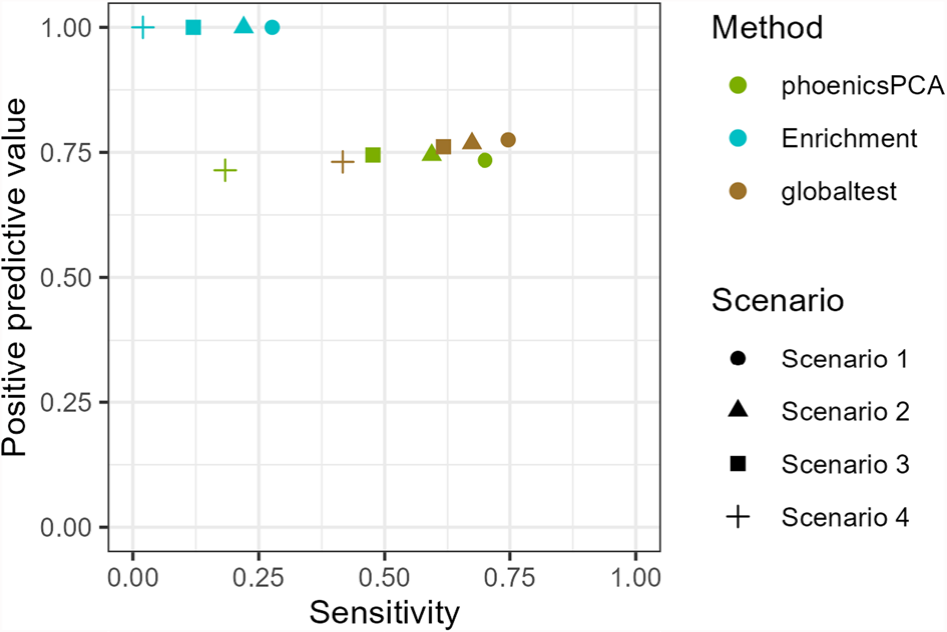
SimulatedH1_Time. Positive predictive value (PPV) and sensitivity. phoenicsMFA and **ktest** are not shown since they detect no positive pathways therefore no PPV can be calculated

Table 7 highlights the fact that phoenicsMFA does not identify any positive pathway across the scenarios (**SimulatedH1_Time**). In contrast, phoenicsPCA and **globaltest** consistently detects a larger number of true positive pathways compared to the other methods, resulting in a higher sensitivity (Fig. 4). Comparing the PPV and sensitivity between these two methods, **globaltest** has better performances than phoenicsPCA, even if **globaltest** detects a larger number of false positive pathways compared to phoenicsPCA when the effect size decreases (Scenarios 3 and 4). The results on the overlapping pathways (Table 7) are consistent with the larger detection power of the time effect of phoenicsPCA and **globaltest**, as they declare positive 45% and 55% of the overlapping pathways, respectively.

**Fig. 5:**
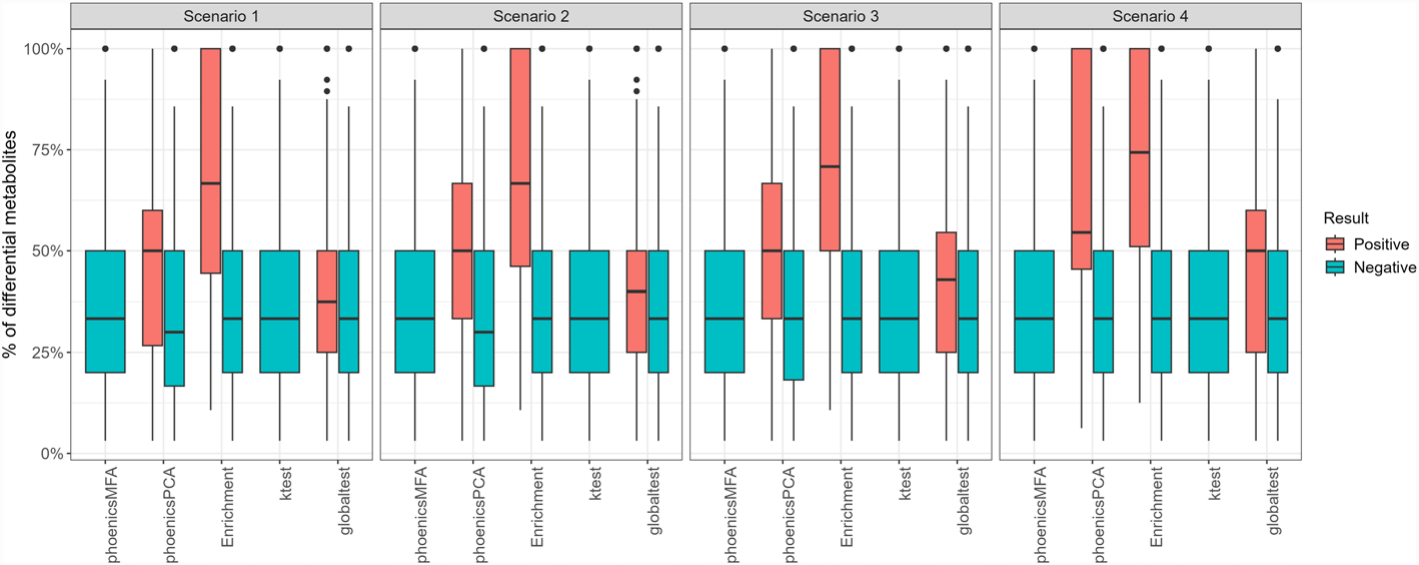
SimulatedH1_Time. Percentage of differential metabolites in the overlapping pathways. Some of these pathways have a percentage of differential metabolites equal to 100% because they are included in a differential pathway

Further investigating the relevance of these overlapping pathways, Fig. 5 shows that positive and negative overlapping pathways identified by **globaltest** have similar percentages of differential metabolites, which makes dubious the relevance of the positive overlapping pathways. In contrast, positive and negative overlapping pathways identified by phoenicsPCA exhibit distinctive percentages of differential metabolites.

**Fig. 6:**
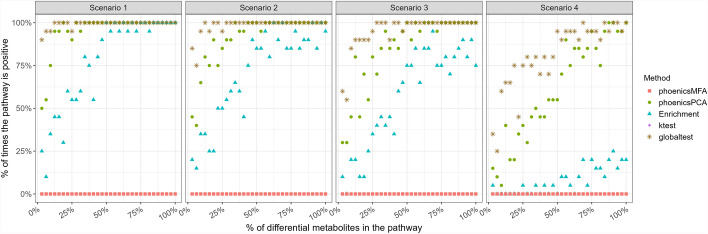
SimulatedVSize_Time. Percentage of times the pathway “ABC transporters” is detected positive over the 20 simulations with respect to the percentage of differential metabolites in the pathway

These results are also confirmed by Fig. 6 that shows the higher percentage of detection for a given percentage of metabolites simulated in the pathway (**SimulatedVSize_Time**).

These findings highlight the larger power of phoenicsPCA compared to the other methods for detecting differential pathways, even in cases where the signal in the data is weaker, and without compromising the correct control of the Type I error. It also confirms that phoenicsMFA is not relevant to test the time effect.

**Table 7:** SimulatedH1_TimeCounts of pathways in each category with semi-synthetic data simulation Scenario 1, 2, 3, and 4. The percentages of pathways corresponding to the counts can be found in Additional File 1: Table S2

		True positive	True negative	False positive	False negative	Positive (overlap)	Negative (overlap)
Scenario 1	phoenicsMFA	0	3071	0	300	0	6429
	phoenicsPCA	210	2995	76	90	2919	3510
	Enrichment	83	3071	0	217	424	6005
	**ktest**	0	3071	0	300	0	6429
	**globaltest**	224	3006	65	76	3542	2887
Scenario 2	phoenicsMFA	0	3071	0	300	0	6429
	phoenicsPCA	178	3010	61	122	2344	4085
	Enrichment	66	3071	0	234	342	6087
	**ktest**	0	3071	0	300	0	6429
	**globaltest**	202	3010	61	98	3239	3190
Scenario 3	phoenicsMFA	0	3071	0	300	0	6429
	phoenicsPCA	143	3022	49	157	1658	4771
	Enrichment	36	3071	0	264	197	6232
	**ktest**	0	3071	0	300	0	6429
	**globaltest**	185	3013	58	115	2850	3179
Scenario 4	phoenicsMFA	0	3071	0	300	0	6429
	phoenicsPCA	55	3049	22	245	561	5868
	Enrichment	6	3071	0	294	42	6387
	**ktest**	0	3071	0	300	0	6429
	**globaltest**	125	3025	46	175	2226	4203

### Testing the condition and the time effects simultaneously

Similarly to the previous simulations, the number of tests (falsely) detected under the null hypothesis (at a significance level of 5%) on dataset **SimulatedH0_ConditionTime** demonstrates that the Type I error is controlled by all the methods when testing for the time and condition effects, except for **globaltest** that does not control the Type I error when testing for the time effect.

The test of the time effect leads to the same conclusions as those obtained when pathways were differential for the time effect only. Complete results can be found in Additional File 1: Table S4, Figs. S1, S2, and S3.

**Fig. 7:**
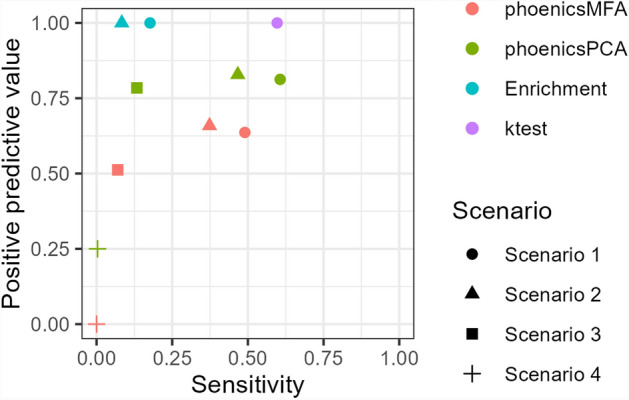
SimulatedH1_ConditionTime. Positive predictive value (PPV) and sensitivity to test the condition effect. Enrichment for Scenarios 3 and 4, **ktest** for Scenarios 2, 3, and 4, and **globaltest** are not shown since they detect no positive pathways therefore no PPV can be calculated

Regarding the test of the condition effect (**SimulatedH1_ConditionTime**), phoenicsPCA has a higher sensitivity than phoenicsMFA and enrichment analysis across scenarios (Fig. 7) since phoenicsPCA detects a larger number of true positive pathways (Table 8). Furthermore, phoenicsPCA exhibits a smaller number of false positives compared to phoenicsMFA so have a higher PPV. Additionally, **ktest** exhibits high sensitivity and PPV in Scenario 1 but detects no more positive when the effect size decreases in the other scenarios.

The study of the percentage of differential metatabolites in the overlapping pathways (Additional File 1: Fig. S4) and the detection power with respect to the percentage of metabolites detected (**SimulatedVSize_ConditionTime**; Additional File 1: Fig. S5) confirm the better detection power of phoenicsPCA for the test of the condition and the time effects, when pathways are differential for both effects. If we compare these results with those obtained when only a condition effect is present, phoenicsPCA seems to be less perturbated in the detection of the effect than other approaches.

**Table 8:** SimulatedH1_ConditionTime. Counts of pathways in each category with semi-synthetic data simulation Scenario 1, 2, 3, and 4 for the test of the condition effect. The percentages of pathways corresponding to the counts can be found in Additional File 1: Table S3

Condition effect							
		True positive	True negative	False positive	False negative	Positive (overlap)	Negative (overlap)
Scenario 1	phoenicsMFA	147	2971	84	153	2381	4063
	phoenicsPCA	182	3013	42	118	2307	4138
	Enrichment	53	3055	0	247	296	6149
	**ktest**	179	3055	0	121	2300	4145
	**globaltest**	0	3055	0	300	0	6445
Scenario 2	phoenicsMFA	112	2997	58	188	1735	4710
	phoenicsPCA	140	3026	29	160	1619	4826
	Enrichment	25	3055	0	275	183	6262
	**ktest**	0	3055	0	300	0	6445
	**globaltest**	0	3055	0	300	0	6445
Scenario 3	phoenicsMFA	21	3035	20	279	326	6119
	phoenicsPCA	40	3044	11	260	519	5962
	Enrichment	0	3055	0	300	4	6441
	**ktest**	0	3055	0	300	0	6445
	**globaltest**	0	3055	0	300	0	6445
Scenario 4	phoenicsMFA	0	3043	12	300	22	6423
	phoenicsPCA	1	3052	3	299	27	6418
	Enrichment	0	3055	0	300	0	6445
	**ktest**	0	3055	0	300	0	6445
	**globaltest**	0	3055	0	300	0	6445

### Analysis of antibiotics effect with phoenics

Table 9 gives an excerpt of detected pathways found by phoenicsPCA and phoenicsMFA for the time and the condition effects, selecting only the pathways that are commented in this section. Full results can be found in Additional File 2: Table S5. For the sake of comparison, the results of the enrichment analysis are also provided in the same table.

Using phoenicsPCA, 72 pathways are found significant for the time effect and one pathway is found significant for the condition effect. phoenicsMFA does not detect any pathway as significant for the time effect but declares 5 pathways as significant for the condition effect. One of these pathways corresponds to the one identified as significant by phoenicsPCA, highlighting the consistency between both methods. The enrichment analysis does not detect any pathway as significant, neither for the time nor for the condition effects.

**Table 9:** Excerpt of the results of the test of time and condition effects on experimental mice antibiotics data with phoenicsPCA, phoenicsMFA and enrichment analysis. The percentage of significant metabolites in the pathways comes from the individual test of the metabolites. Full result table is available in Additional File 2: Table S5

Pathway	phoenicsPCA	phoenicsMFA	Enrichment	% of significant metabolites (Time)	% of significant metabolites (Condition)	Number of quantified metabolites in the pathway
Phenylalanine, tyrosine and tryptophan biosynthesis	Time	-	-	67%	0%	3
Primary bile acid biosynthesis	Time	-	-	50%	0%	2
Bile secretion	Time	-	-	50%	0%	4
Tryptophan metabolism	Time	-	-	50%	0%	4
Valine, leucine and isoleucine degradation	Time	-	-	86%	0%	7
Valine, leucine and isoleucine biosynthesis	Time	-	-	38%	0%	8
Choline metabolism in cancer	Time, Condition	Condition	-	0%	0%	2
Butanoate metabolism	-	Condition	-	64%	0%	11

**Fig. 8:**
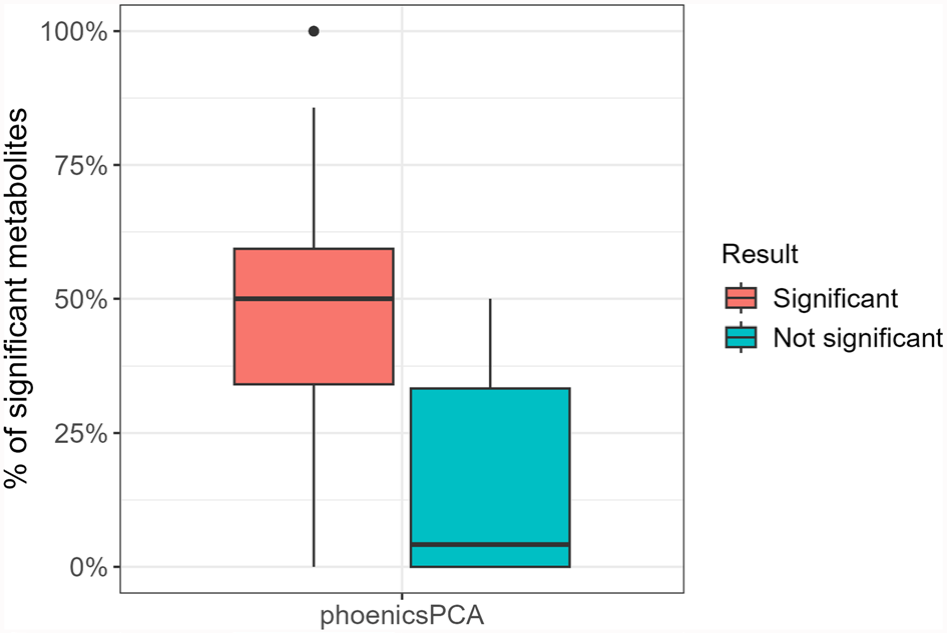
Percentage of significant metabolites for the time effect with respect to the pathway status (significant or not for phoenicsPCA)

The percentage of significant metabolites in the pathways for the time effect, obtained by the analysis of each metabolite individually by phoenicsPCA, is presented in Fig. 8 and Table 9. As expected, the significant pathways contain a larger percentage of significant metabolites than the non-significant pathways. The median percentage of significant metabolites in the non-significant pathways detected by phoenicsPCA is low. Note that for both effects and all pathways on a standard desktop computer, phoenicsPCA lasts 60 s, phoenicsMFA 63 s, and the enrichment method 44 s.

In addition, some pathways are significant for the time effect with phoenicsPCA but not with the enrichment analysis due to the small number of significant metabolites in the individual metabolite analysis. This underscores a major difference between phoenicsPCA and the enrichment analysis: phoenicsPCA performs a multivariate analysis, considering the correlations between metabolites in a pathway, thus providing a better representation of the overall pathway dynamics. It is thus able to detect differential pathways with a large proportion of metabolites presenting small size effects and non-significant but consistent variations. For example, the “Choline metabolism” pathway is found to be significant, even though it contains only two metabolites, neither of which are individually significant. This highlights the strength of the methodology and phoenics’ ability to detect subtle but accumulated pathway modifications, even in small pathways. In contrast, the enrichment analysis relies only on the number of significant metabolites obtained in independent analyses, without considering the correlation between metabolites. It is thus unable to detect pathways cumulating several metabolites with small size and consistent effects. Furthermore, if both ORA and phoenics include a two-step multiple testing correction, the first step is performed at metabolite level for ORA, whereas it is less stringent because performed at dimension reduction level for phoenics. However, additional analyses not shown in the article showed that, despite not statistically valid, removing the multiple testing correction at metabolomic level in ORA analysis does not change the conclusion of the comparison.

From a biological point of view, Choo et al. [32] did not study the longitudinal effect of antibiotics on metabolome so our results can not be directly compared to what they did. Nevertheless, several pathways identified by phoenicsPCA are linked to functions of the gut metabolome. For instance, a major branch of the “Tryptophan metabolism” pathway is the kynurenine pathway, which is important for immunomodulatory microbiota metabolites [42, 43]. The pathways “Primary bile acids biosynthesis” and “Bile secretion” are identified as significant with phoenicsPCA. These findings are supported by Mars et al. [37], de Aguiar Vallim et al. [44], and Liu et al. [45] who stated that bile acids are common fecal metabolites that have several functions in the gut microbiota, including the regulation of the gut microbiota composition. Furthermore, Just et al. [46] also observed changes in the metabolome induced by bile acids supplementation in the diet of mice. The pathway “Phenylalanine, tyrosine and tryptophan biosynthesis” is also found significant and, as mentioned by Liu et al. [45], phenylalanine and tyrosine can be synthesized by the gut microbiota. Finally, the pathways “Valine, leucine and isoleucine degradation” and “Valine, leucine and isoleucine biosynthesis” have already been identified in mice feces-based study by Zeng et al. [47].

For the condition effect, we observe a larger number of significant pathways with phoenicsMFA than with phoenicsPCA, whereas these pathways are also significant for the time effect. Investigating these additional significant pathways identified by phoenicsMFA, we find that they contain a larger number of metabolites compared to the non-significant pathways. The analysis of each metabolite individually results in no significant metabolite. However, the pathway “Choline metabolism in cancer” is significant with phoenicsPCA, as it is also the case for the time effect, and with phoenicsMFA. This is consistent with the fact that Liu et al. [48] previously reported that choline metabolism is perturbed by some antibiotics, including vancomycin that has been used in the experiment. In addition, the pathway “Butanoate metabolism” is significant with phoenicsMFA which is consistent with the fact that butanoate, also named butyrate, is produced in the colon and is known to play an important role in the gut health [49, 50]. Moreover, Yap et al. [51] observed a change in the amount of butyrate on mice treated with vancomycin compared to non-treated mice. Thus, pathways identified by phoenicsPCA and phoenicsMFA play roles connected to the gut metabolome functions.

### Analysis of irritable bowel syndrome with phoenics

An excerpt of significant pathways found by phoenicsPCA, phoenicsMFA, and compared to enrichment analysis, for the time and the condition effect is given in Table 10. It includes only the pathways commented in this section, full results can be found in Additional File 3: Table S6.

For the time effect, two pathways are found significant by phoenicsPCA, whereas no pathway is found significant by phoenicsMFA and enrichment analysis. Mars et al. [37] did not study the longitudinal effect of the irritable bowel syndrome on metabolome, except for one metabolite. However, phoenicsPCA found significant the pathway “Ferroptosis” for the time effect. Several studies [52–54] have shown the implication of ferroptosis, a form of cell death induced by an excess of iron, in irritable bowel syndrome. This pathway was not identified in the study of Mars et al. [37], highlighting the benefits of performing longitudinal analysis with **phoenics** to identify new relevant pathways.

For the condition effect, 72 pathways are found significant by phoenicsPCA, 56 by phoenicsMFA and four by the enrichment analysis. For the condition effect, the pathway “Purine metabolism” is detected exclusively by phoenicsPCA. This pathway was mentioned by Mars et al. [37] as potentially being involved in the pathophysiology of irritable bowel syndrome. The metabolite “hypoxanthine” is contained in this pathway and was measured at lower levels in IBS-C samples by Mars et al. [37]. The pathway “Carbohydrate digestion and absorption” is detected by all methods. It is confirmed by the presence of the metabolites “acetate” and “butanoic acid” (also known as “butyrate”) in the pathway, which were identified by Mars et al. [37] as being present at lower abundance in IBS-C samples compared to healthy controls. In addition, Mars et al. [37] also identified significant differences between the IBS-C and control samples in a genomic region associated with butyrate production. This result is confirmed by [55, 56], which showed that poor absorption and digestion of carbohydrate can induce symptoms of irritable bowel syndrome. In conclusion, **phoenics** was able to identify relevant pathways for the analysis of irritable bowel syndrome.

**Table 10:** Excerpt of the results of the test of time and condition effects on experimental human irritable bowel syndrome data with phoenicsPCA, phoenicsMFA and enrichment analysis. The percentage of significant metabolites in the pathways comes from the individual test of the metabolites. Full result table is available in Additional File 3: Table S6

Pathway	phoenicsPCA	phoenicsMFA	Enrichment	% of significant metabolites (Time)	% of significant metabolites (Condition)	Number of quantified metabolites in the pathway
Ferroptosis	Time, Condition	Condition	-	50%	25%	4
Purine metabolism	Condition	-	-	0%	17%	12
Carbohydrate digestion and absorption	Condition	Condition	Condition	0%	100%	5

## Conclusion

The article presents an approach to perform a differential and longitudinal analysis of metabolic pathways, available in the R package **phoenics**. The method is based on factor analysis and mixed linear models to perform the analysis at a pathway level and it does not require prior analysis of metabolites. Two versions are available, respectively based on PCA and MFA pathway summaries. Since MFA is a multi-table analysis method designed to analyze tables with matched individuals, it is a natural way to extract multivariate signal from datasets with multiple measurements (e.g., time in multivariate longitudinal analyses) and can further be used to test any fixed effect except for the one structuring the multiple measurements. Indeed, the effect associated with repeated measurements (e.g., the time) is lost during MFA (and thus can not be tested). This effect is, however, properly captured by the PCA version of **phoenics**. Note that two time points are, in theory, sufficient to fit a simple mixed effect model. However, this number of time points might be too little for the estimation of the random effect to be of good quality. For a better quality of the method results, we recommend using phoenics with three or more time points.

The application of **phoenics** on semi-synthetic data showed that the method detects differential pathways with a higher power than existing methods while properly controlling the Type I error rate. On a real datasets, **phoenics** methods were able to identify pathways relevant to describe the effect of antibiotics in mice and of irritable bowel syndrome in human on feces metabolomics.

The method is generic and flexible with respect to pathway or to metabolomics data types: The illustration in the article has focused on KEGG pathways and NMR datasets but the method is generic enough to accomodate other pathway databases or metabolomic quantifications coming for any other acquisition technique (such as mass spectrometry). It is known that pathway choice can strongly impact the results of the analysis [57]. The choice of KEGG is the most commonly done choice but certain KEGG pathways can be so large to the point where their utility is sometimes questioned. Investigating the most appropriate pathway database or combining results from different databases with phoenics might thus be an interesting issue to address in future works. Finally, in future works, we also intend to extend this approach to the integration of longitudinal multi-omics data.

## Supplementary Information

Supplementary file 1: Figure S1. Positive predictive value (PPV) and sensitivity for the test of the time effect in simulation SimulatedH1_ConditionTime. Figure S2. Percentage of differential metabolites for the time effect in the overlapping pathways in simulation SimulatedH1_ConditionTime. Table S1. Results of the test of the condition effect in simulation SimulatedH1_Condition. Table S2. Results of the test of the time effect in simulation SimulatedH1_Time. Table S3. Results of the test of the condition effect in simulation SimulatedH1_ConditionTime. Table S4. Results of the test of the time effect in simulation SimulatedH1_ConditionTime. Figure S3. Percentage of times the pathway “ABC transporters” is detected positive for the test of the time effect in simulation SimulatedVSize_ConditionTime. Figure S4. Percentage of differential metabolites for the condition effect in the overlapping pathways in simulation SimulatedH1_ConditionTime. Figure S5. Percentage of times the pathway “ABC transporters” is detected positive for the test of the condition effect in simulation SimulatedVSize_ConditionTime. (pdf file).

Supplementary file 2: Table S5. Full results of the analysis of antibiotics with phoenicsPCA, phoenicsMFA, and enrichment analysis (xls file).

Supplementary file 3: Table S6. Full results of the analysis of irritable bowel syndrome with phoenicsPCA, phoenicsMFA, and enrichment analysis (xls file).

## Data Availability

The dataset from Choo et al. [32] is available at https://www.ebi.ac.uk/metabolights/editor/MTBLS422. The dataset from Mars et al. [37] is available at https://www.ebi.ac.uk/metabolights/editor/MTBLS1396. All the scripts used to perform the analyses of this article are available at https://forgemia.inra.fr/panoramics/rlib_bmcbioinformatics_2024.

## Footnotes

1 https://CRAN.R-project.org/package=phoenics

## References

[1] Fiehn O. Metabolomics—the link between genotypes and phenotypes. Plant Mol Biol. 2002;48(1/2):155–71. 10.1023/a:1013713905833. 11860207

[2] Johnson CH, Ivanisevic J, Siuzdak G. Metabolomics: beyond biomarkers and towards mechanisms. Nat Rev Mol Cell Biol. 2016;17(7):451–9. 10.1038/nrm.2016.25. 26979502 10.1038/nrm.2016.25 PMC5729912

[3] Larive CK, Barding GA, Dinges MM. NMR spectroscopy for metabolomics and metabolic profiling. Anal Chem. 2014;87(1):133–46. 10.1021/ac504075g. 25375201 10.1021/ac504075g

[4] Trivedi KDA, Hollywood K, Goodacre R. Metabolomics for the masses: the future of metabolomics in a personalized world. New Horiz Trans Med. 2017;3(6):294. 10.1016/j.nhtm.2017.06.001. 10.1016/j.nhtm.2017.06.001 PMC5653644 29094062

[5] Miyamoto S, Taylor S, Barupal D, Taguchi A, Wohlgemuth G, Wikoff W, Yoneda K, Gandara D, Hanash S, Kim K, Fiehn O. Systemic metabolomic changes in blood samples of lung cancer patients identified by gas chromatography time-of-flight mass spectrometry. Metabolites. 2015;5(2):192–210. 10.3390/metabo5020192. 25859693 10.3390/metabo5020192 PMC4495369

[6] Rubingh CM, Bijlsma S, Jellema RH, Overkamp KM, Werf MJ, Smilde AK. Analyzing longitudinal microbial metabolomics data. J Proteome Res. 2009;8(9):4319–27. 10.1021/pr900126e. 19624157 10.1021/pr900126e

[7] Sindelar M, Stancliffe E, Schwaiger-Haber M, Anbukumar DS, Adkins-Travis K, Goss CW, O’Halloran JA, Mudd PA, Liu W-C, Albrecht RA, García-Sastre A, Shriver LP, Patti GJ. Longitudinal metabolomics of human plasma reveals prognostic markers of COVID-19 disease severity. Cell Rep Med. 2021;2(8): 100369. 10.1016/j.xcrm.2021.100369. 34308390 10.1016/j.xcrm.2021.100369 PMC8292035

[8] Mäkinen V-P, Karsikas M, Kettunen J, Lehtimäki T, Kähönen M, Viikari J, Perola M, Salomaa V, Järvelin M-R, Raitakari OT, Ala-Korpela M. Longitudinal profiling of metabolic ageing trends in two population cohorts of young adults. Int J Epidemiol. 2022;51(6):1970–83. 10.1093/ije/dyac062. 35441226 10.1093/ije/dyac062

[9] Molenberghs G, Verbeke G. Linear Mixed Models for Longitudinal Data. New-York: Springer; 2000.

[10] Martin M, Govaerts B. LiMM-PCA: combining ASCA+ and linear mixed models to analyse high-dimensional designed data. J Chemom. 2020;34(6):3232. 10.1002/cem.3232.

[11] Khatri P, Sirota M, Butte AJ. Ten years of pathway analysis: Current approaches and outstanding challenges. PLoS Comput Biol. 2012;8(2):1002375. 10.1371/journal.pcbi.1002375. 10.1371/journal.pcbi.1002375 PMC3285573 22383865

[12] Maleki F, Ovens K, Hogan DJ, Kusalik AJ. Gene set analysis: challenges, opportunities and future research. Front Genet. 2020;11:654. 10.3389/fgene.2020.00654. 32695141 10.3389/fgene.2020.00654 PMC7339292

[13] Mootha VK, Lindgren CM, Eriksson K-F, Subramanian A, Sihag S, Lehar J, Puigserver P, Carlsson E, Ridderstråle Laurila E, Houstis N, Daly MJ, Patterson N, Mesirov JP, Golub TR, Tamayo P, Spiegelman B, Lander ES, Hirschhorn JN, Altshuler D, Groop LC. PGC-1α-responsive genes involved in oxidative phosphorylation are coordinately downregulated in human diabetes. Nat Genet. 2003;34:267–73. 10.1038/ng1180. 12808457 10.1038/ng1180

[14] Tian L, Greenberg SA, Kong SW, Altschuler J, Kohane IS, Park PJ. Discovering statistically significant pathways in expression profiling studies. Proc Natl Acad Sci USA. 2005;102(38):13544–9. 10.1073/pnas.0506577102. 16174746 10.1073/pnas.0506577102 PMC1200092

[15] Kim S-Y, Volsky DJ. PAGE: parametric analysis of gene set enrichment. BMC Bioinfo. 2005;6:144. 10.1186/1471-2105-6-144. 10.1186/1471-2105-6-144 PMC1183189 15941488

[16] Subramanian A, Tamayo P, Mootha VK, Mukherjee S, Ebert BL, Gillette MA, Paulovich A, Pomeroy SL, Golub TR, Lander ES, Mesirov JP. Gene set enrichment analysis: a knowledge-based approach for interpreting genome-wide expression profiles. Proc Natl Acad Sci. 2005;102(43):15545–50. 10.1073/pnas.0506580102. 16199517 10.1073/pnas.0506580102 PMC1239896

[17] Irizarry RA, Wang C, Zhou Y, Speed TP. Gene set enrichment analysis made simple. Stat Methods Med Res. 2009;18(6):565–75. 10.1177/0962280209351908. 20048385 10.1177/0962280209351908 PMC3134237

[18] Jiang Z, Gentleman R. Extensions to gene set enrichment. Bioinformatics. 2007;23(3):306–13. 10.1093/bioinformatics/btl599. 17127676 10.1093/bioinformatics/btl599

[19] Wieder C, Cooke J, Frainay C, Poupin N, Bowler R, Jourdan F, Kechris KJ, Lai RP, Ebbels T. PathIntegrate: multivariate modelling approaches for pathway-based multi-omics data integration. PLoS Comput Biol. 2024;20(3):1011814. 10.1371/journal.pcbi.1011814. 10.1371/journal.pcbi.1011814 PMC10994553 38527092

[20] Wieder C, Lai RPJ, Ebbels TMD. Single sample pathway analysis in metabolomics: performance evaluation and application. BMC Bioinf. 2022;23:481. 10.1186/s12859-022-05005-1. 10.1186/s12859-022-05005-1 PMC9664704 36376837

[21] Goeman JJ, Geer SA, Kort F, Houwelingen HC. A global test for groups of genes: testing association with a clinical outcome. Bioinformatics. 2004;20(1):93–9. 10.1093/bioinformatics/btg382. 14693814 10.1093/bioinformatics/btg382

[22] Kong SW, Pu WT, Park PJ. A multivariate approach for integrating genome-wide expression data and biological knowledge. Bioinformatics. 2006;22(19):2373–80. 10.1093/bioinformatics/btl401. 16877751 10.1093/bioinformatics/btl401 PMC2813864

[23] Hotelling H. Relations between two sets of variates. Biometrika. 1936;28(3–4):321–77. 10.1093/biomet/28.3-4.321.

[24] Ozier-Lafontaine A, Fourneaux C, Durif G, Arsenteva P, Vallot C, Gandrillon O, Gonin-Giraud S, Michel B, Picard F. Kernel-based testing for single-cell differential analysis. Genome Biol. 2024;25:114. 10.1186/s13059-024-03255-1. 38702740 10.1186/s13059-024-03255-1 PMC11069218

[25] Wieder C, Frainay C, Poupin N, Rodríguez-Mier P, Vinson F, Cooke J, Lai RP, Bundy JG, Jourdan F, Ebbels T. Pathway analysis in metabolomics: recommendations for the use of over-representation analysis. PLoS Comput Biol. 2021;17(9):1009105. 10.1371/journal.pcbi.1009105. 10.1371/journal.pcbi.1009105 PMC8448349 34492007

[26] Simes RJ. An improved Bonferroni procedure for multiple tests of significance. Biometrika. 1986;73(3):751–4. 10.1093/biomet/73.3.751.

[27] Goeman JJ, Solari A. Multiple hypothesis testing in genomics. Stat Med. 2014;33(11):1946–78. 10.1002/sim.6082. 24399688 10.1002/sim.6082

[28] Rødland EA. Simes’ procedure is ‘valid on average’. Biometrika. 2006;93(3):742–6. 10.1093/biomet/93.3.742.

[29] Benjamini Y, Hochberg Y. Controlling the false discovery rate: a practical and powerful approach to multiple testing. J R Stat Soc Ser B Stat Methodol. 1995;57(1):289–300. 10.1111/j.2517-6161.1995.tb02031.x.

[30] Benjamini Y, Yekutieli D. The control of the false discovery rate in multiple testing under dependency. Ann Stat. 2001. 10.1214/aos/1013699998.

[31] Escofier B, Pagès J. Multiple factor analysis (AFMULT package). Comput Stat Data Anal. 1994;18(1):121–40. 10.1016/0167-9473(94)90135-x.

[32] Choo JM, Kanno T, Zain NMM, Leong LEX, Abell GCJ, Keeble JE, Bruce KD, Mason AJ, Rogers GB. Divergent relationships between fecal microbiota and metabolome following distinct antibiotic-induced disruptions. mSphere. 2017. 10.1128/msphere.00005-17. 28194448 10.1128/mSphere.00005-17 PMC5299068

[33] Yurekten O, Payne T, Tejera N, Amaladoss FX, Martin C, Williams M, O’Donovan C. MetaboLights: open data repository for metabolomics. Nucleic Acids Res. 2023;52(D1):640–6. 10.1093/nar/gkad1045. 10.1093/nar/gkad1045 PMC10767962 37971328

[34] Lefort G, Liaubet L, Canlet C, Tardivel P, Père M-C, Quesnel H, Paris A, Iannuccelli N, Vialaneix N, Servien R. ASICS: an R package for a whole analysis workflow of 1D 1H NMR spectra. Bioinformatics. 2019;35(21):4356–63. 10.1093/bioinformatics/btz248. 30977816 10.1093/bioinformatics/btz248

[35] Tenenbaum D, Maintainer BP KEGGREST: Client-side REST Access to the Kyoto Encyclopedia of Genes and Genomes (KEGG). 2022; R package version 1.38.0

[36] Kanehisa M, Goto S. KEGG: kyoto encyclopedia of genes and genomes. Nucleic Acids Res. 2000;28(1):27–30. 10.1093/nar/28.1.27. 10592173 10.1093/nar/28.1.27 PMC102409

[37] Mars R, Yang Y, Ward T, Houtti M, Priya S, Lekatz HR, Tang X, Sun Z, Kalari KR, Korem T, Bhattarai Y, Zheng T, Bar N, Frost G, Johnson AJ, Treuren W, Han S, Ordog T, Grover M, Sonnenburg J, D’Amato M, Camilleri M, Elinav E, Segal E, Blekhman R, Farrugia G, Swann JR, Knights D, Kashyap PC. Longitudinal multi-omics rrveals subset-specific mechanisms underlying irritable bowel syndrome. Cell. 2020;182(6):1460–147317. 10.1016/j.cell.2020.08.007. 32916129 10.1016/j.cell.2020.08.007 PMC8109273

[38] Rosato A, Tenori L, Cascante M, De Atauri Carulla PR, Santos VAP, Saccenti E. From correlation to causation: analysis of metabolomics data using systems biology approaches. Metabolomics. 2018. 10.1007/s11306-018-1335-y. 29503602 10.1007/s11306-018-1335-y PMC5829120

[39] Harchaoui Z, Vallet F, Lung-Yut-Fong A, Cappe O. A regularized kernel-based approach to unsupervised audio segmentation. In: 2009 IEEE International Conference on Acoustics, Speech and Signal Processing, pp. 1665–1668. IEEE, Taipei, Taiwan 2009; 10.1109/icassp.2009.4959921.

[40] Hotelling H. The generalization of student’s ratio. Ann Math Stat. 1931;2(3):360–78. 10.1214/aoms/1177732979.

[41] Nelder JA, Wedderburn RWM. Generalized linear models. J Royal Stat Soc Series A. 1972;135(3):370. 10.2307/2344614.

[42] Krishnan S, Alden N, Lee K. Pathways and functions of gut microbiota metabolism impacting host physiology. Curr Opin Biotechnol. 2015;36:137–45. 10.1016/j.copbio.2015.08.015. 26340103 10.1016/j.copbio.2015.08.015 PMC4688195

[43] Tsuji A, Ikeda Y, Yoshikawa S, Taniguchi K, Sawamura H, Morikawa S, Nakashima M, Asai T, Matsuda S. The tryptophan and kynurenine pathway involved in the development of immune-related diseases. Int J Mol Sci. 2023;24(6):5742. 10.3390/ijms24065742. 36982811 10.3390/ijms24065742 PMC10051340

[44] Aguiar Vallim TQ, Tarling EJ, Edwards PA. Pleiotropic roles of bile acids in metabolism. Cell Metab. 2013;17(5):657–69. 10.1016/j.cmet.2013.03.013. 23602448 10.1016/j.cmet.2013.03.013 PMC3654004

[45] Liu J, Tan Y, Cheng H, Zhang D, Feng W, Peng C. Functions of gut microbiota metabolites, current status and future perspectives. Aging Dis. 2022;13(4):1106. 35855347 10.14336/AD.2022.0104 PMC9286904

[46] Just S, Mondot S, Ecker J, Wegner K, Rath E, Gau L, Streidl T, Hery-Arnaud G, Schmidt S, Lesker TR, Bieth V, Dunkel A, Strowig T, Hofmann T, Haller D, Liebisch G, Gérard P, Rohn S, Lepage P, Clavel T. The gut microbiota drives the impact of bile acids and fat source in diet on mouse metabolism. Microbiome. 2018;6:10–1. 10.1186/s40168-018-0510-8. 30071904 10.1186/s40168-018-0510-8 PMC6091023

[47] Zeng S-L, Li S-Z, Xiao P-T, Cai Y-Y, Chu C, Chen B-Z, Li P, Li J, Liu E-H. Citrus polymethoxyflavones attenuate metabolic syndrome by regulating gut microbiome and amino acid metabolism. Sci Adv. 2020. 10.1126/sciadv.aax6208. 31922003 10.1126/sciadv.aax6208 PMC6941918

[48] Liu Z, Xia B, Saric J, Utzinger J, Holmes E, Keiser J, Li JV. Effects of vancomycin and ciprofloxacin on the NMRI mouse metabolism. J Proteome Res. 2018;17(10):3565–73. 10.1021/acs.jproteome.8b00583. 30183313 10.1021/acs.jproteome.8b00583

[49] Louis P, Flint HJ. Diversity, metabolism and microbial ecology of butyrate-producing bacteria from the human large intestine. FEMS Microbiol Lett. 2009;294(1):1–8. 10.1111/j.1574-6968.2009.01514.x. 19222573 10.1111/j.1574-6968.2009.01514.x

[50] Hamer HM, Jonkers D, Venema K, Vanhoutvin S, Troost FJ, Brummer R-J. Review article: the role of butyrate on colonic function. Aliment Pharmacol Ther. 2007;27(2):104–19. 10.1111/j.1365-2036.2007.03562.x. 17973645 10.1111/j.1365-2036.2007.03562.x

[51] Yap I, Li JV, Saric J, Martin F-P, Davies H, Wang Y, Wilson ID, Nicholson JK, Utzinger J, Marchesi JR, Holmes E. Metabonomic and microbiological analysis of the dynamic effect of vancomycin-induced gut microbiota modification in the mouse. J Proteome Res. 2008;7(9):3718–28. 10.1021/pr700864x. 18698804 10.1021/pr700864x

[52] Xu M, Tao J, Yang Y, Tan S, Liu H, Jiang J, Zheng F, Wu B. Ferroptosis involves in intestinal epithelial cell death in ulcerative colitis. Cell Death Dis. 2020. 10.1038/s41419-020-2299-1. 32015337 10.1038/s41419-020-2299-1 PMC6997394

[53] Chen Y, Zhang P, Chen W, Chen G. Ferroptosis mediated DSS-induced ulcerative colitis associated with Nrf2/HO-1 signaling pathway. Immunol Lett. 2020;225:9–15. 10.1016/j.imlet.2020.06.005. 32540488 10.1016/j.imlet.2020.06.005

[54] Xu J, Liu S, Cui Z, Wang X, Ning T, Wang T, Zhang N, Xie S, Min L, Zhang S, Liang C, Zhu S. Ferrostatin-1 alleviated TNBS induced colitis via the inhibition of ferroptosis. Biochem Biophys Res Commun. 2021;573:48–54. 10.1016/j.bbrc.2021.08.018. 34388454 10.1016/j.bbrc.2021.08.018

[55] Major G, Pritchard S, Murray K, Alappadan JP, Hoad CL, Marciani L, Gowland P, Spiller R. Colon hypersensitivity to distension, rather than excessive gas production, produces carbohydrate-related symptoms in individuals with irritable bowel syndrome. Gastroenterology. 2017;152(1):124–1332. 10.1053/j.gastro.2016.09.062. 27746233 10.1053/j.gastro.2016.09.062

[56] Shepherd S, Parker F, Muir J, Gibson P. Dietary triggers of abdominal symptoms in patients with irritable bowel syndrome: randomized placebo-controlled evidence. Clin Gastroenterol Hepatol. 2008;6(7):765–71. 10.1016/j.cgh.2008.02.058. 18456565 10.1016/j.cgh.2008.02.058

[57] Mubeen S, Hoyt CT, Gemünd A, Hofmann-Apitius M, Fröhlich H, Domingo-Fernández D. The impact of pathway database choice on statistical enrichment analysis and predictive modeling. Front Genet. 2019;10:1203. 10.3389/fgene.2019.01203. 31824580 10.3389/fgene.2019.01203 PMC6883970

