# Supplementary material for: Phoenics: a novel statistical approach for longitudinal metabolomic pathway analysis
Source: BMC Bioinformatics. 2025 Apr 16;26:105. doi: 10.1186/s12859-025-06118-z (PMC12001596; doi:10.1186/s12859-025-06118-z)
Supplement: Supplementary file 1 — Figure S1. Positive predictive value (PPV) and sensitivity for the test of the time effect in simulation SimulatedH1_ConditionTime. Figure S2. Percentage of differential metabolites for the time effect in the overlapping pathways in simulation SimulatedH1_ConditionTime. Table S1. Results of the test of the condition effect in simulation SimulatedH1_Condition. Table S2. Results of the test of the time effect in simulation SimulatedH1_Time. Table S3. Results of the test of the condition effect in simulation SimulatedH1_ConditionTime. Table S4. Results of the test of the time effect in simulation SimulatedH1_ConditionTime. Figure S3. Percentage of times the pathway “ABC transporters” is detected positive for the test of the time effect in simulation SimulatedVSize_ConditionTime. Figure S4. Percentage of differential metabolites for the condition effect in the overlapping pathways in simulation SimulatedH1_ConditionTime. Figure S5. Percentage of times the pathway “ABC transporters” is detected positive for the test of the condition effect in simulation SimulatedVSize_ConditionTime. (pdf file). [file 12859_2025_6118_MOESM1_ESM.pdf]

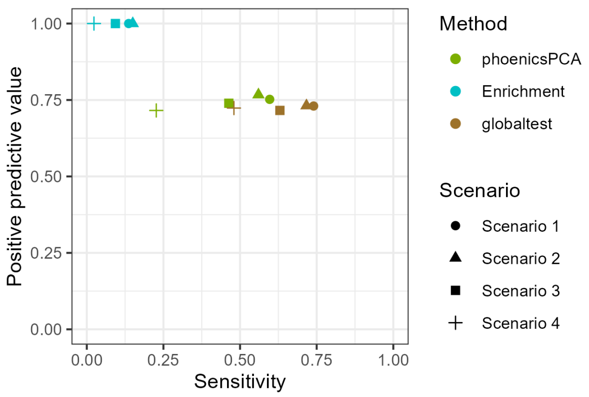

Fig. S1 SimulatedH1_ConditionTime. Positive predictive value (PPV) and sensitivity to test the time effect. phoenicsMFA and **ktest** do not appear in the figure since they detect no positive pathways therefore no PPV can be calculated.

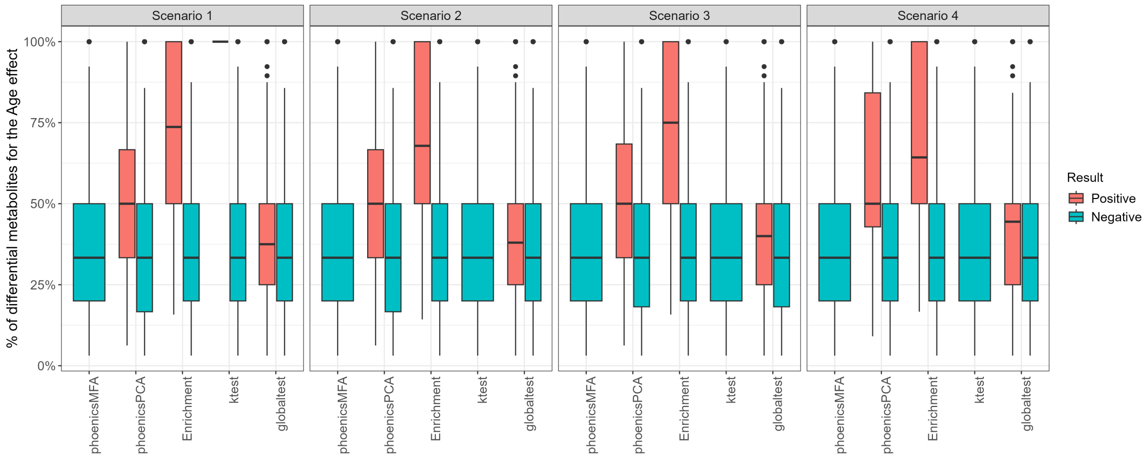

Fig. S2 SimulatedH1_ConditionTime. Percentage of differential metabolites for the time effect in the overlapping pathways. Some of these pathways have a percentage of differential metabolites equal to 100% because they are included in a differential pathway.

Table S1 SimulatedH1_Condition. Counts and percentage of pathways in each category with semi-synthetic data simulation Scenario 1, 2, and 3.

		True positive	True negative	False positive	False negative	Positive (overlap)	Negative (overlap)
Scenario 1	phoenicsMFA	251 (88%)	2789 (100%)	0 (0%)	33 (12%)	3065 (51%)	2962 (49%)
	phoenicsPCA	239 (80%)	3153 (99%)	22 (1%)	61 (20%)	2762 (44%)	3563 (56%)
	Enrichment	127 (42%)	3175 (100%)	0 (0%)	173 (58%)	584 (9%)	5741 (91%)
	**ktest**	38 (13%)	3175 (100%)	0 (0%)	262 (87%)	199 (3%)	6126 (97%)
	**globaltest**	0 (0%)	3175 (100%)	0 (0%)	300 (100%)	0 (0%)	6325 (100%)
Scenario 2	phoenicsMFA	228 (80%)	2789 (100%)	0 (0%)	56 (20%)	2327 (39%)	3700 (61%)
	phoenicsPCA	180 (60%)	3161 (100%)	14 (0%)	120 (40%)	1886 (30%)	4439 (70%)
	Enrichment	68 (23%)	3175 (100%)	0 (0%)	232 (77%)	353 (6%)	5972 (94%)
	**ktest**	0 (0%)	3175 (100%)	0 (0%)	300 (100%)	0 (0%)	6325 (100%)
	**globaltest**	0 (0%)	3175 (100%)	0 (0%)	300 (100%)	0 (0%)	6325 (100%)
Scenario 3	phoenicsMFA	163 (57%)	2789 (100%)	0 (0%)	121 (43%)	1631 (27%)	4396 (73%)
	phoenicsPCA	85 (28%)	3172 (100%)	3 (0%)	215 (72%)	1018 (16%)	5307 (84%)
	Enrichment	1 (0%)	3175 (100%)	0 (0%)	299 (100%)	2 (0%)	6323 (100%)
	**ktest**	0 (0%)	3175 (100%)	0 (0%)	300 (100%)	0 (0%)	6325 (100%)
	**globaltest**	0 (0%)	3175 (100%)	0 (0%)	300 (100%)	0 (0%)	6325 (100%)

Table S2 SimulatedH1_Time. Counts and percentages of pathways in each category with semi-synthetic data simulation Scenario 1, 2, 3, and 4.

		True positive	True negative	False positive	False negative	Positive (overlap)	Negative (overlap)
Scenario 1	phoenicsMFA	0 (0%)	3071 (100%)	0 (0%)	300 (100%)	0 (0%)	6429 (100%)
	phoenicsPCA	210 (70%)	2995 (98%)	76 (2%)	90 (30%)	2919 (45%)	3510 (55%)
	Enrichment	83 (28%)	3071 (100%)	0 (0%)	217 (72%)	424 (7%)	6005 (93%)
	**ktest**	0 (0%)	3071 (100%)	0 (0%)	300 (100%)	0 (0%)	6429 (100%)
	**globaltest**	224 (75%)	3006 (98%)	65 (2%)	76 (25%)	3542 (55%)	2887 (45%)
Scenario 2	phoenicsMFA	0 (0%)	3071 (100%)	0 (0%)	300 (100%)	0 (0%)	6429 (100%)
	phoenicsPCA	178 (59%)	3010 (98%)	61 (2%)	122 (41%)	2344 (36%)	4085 (64%)
	Enrichment	66 (22%)	3071 (100%)	0 (0%)	234 (78%)	342 (5%)	6087 (95%)
	**ktest**	0 (0%)	3071 (100%)	0 (0%)	300 (100%)	0 (0%)	6429 (100%)
	**globaltest**	202 (67%)	3010 (98%)	61 (2%)	98 (33%)	3239 (50%)	3190 (50%)
Scenario 3	phoenicsMFA	0 (0%)	3071 (100%)	0 (0%)	300 (100%)	0 (0%)	6429 (100%)
	phoenicsPCA	143 (48%)	3022 (98%)	49 (2%)	157 (52%)	1658 (26%)	4771 (74%)
	Enrichment	36 (12%)	3071 (100%)	0 (0%)	264 (88%)	197 (3%)	6232 (97%)
	**ktest**	0 (0%)	3071 (100%)	0 (0%)	300 (100%)	0 (0%)	6429 (100%)
	**globaltest**	185 (62%)	3013 (98%)	58 (2%)	115 (38%)	2850 (44%)	3179 (49%)
Scenario 4	phoenicsMFA	0 (0%)	3071 (100%)	0 (0%)	300 (100%)	0 (0%)	6429 (100%)
	phoenicsPCA	55 (18%)	3049 (99%)	22 (1%)	245 (82%)	561 (9%)	5868 (91%)
	Enrichment	6 (2%)	3071 (100%)	0 (0%)	294 (98%)	42 (1%)	6387 (99%)
	**ktest**	0 (0%)	3071 (100%)	0 (0%)	300 (100%)	0 (0%)	6429 (100%)
	**globaltest**	125 (42%)	3025 (99%)	46 (1%)	175 (58%)	2226 (35%)	4203 (65%)

Table S3 SimulatedH1_ConditionTime. Counts and percentages of pathways in each category with semi-synthetic data simulation Scenario 1, 2, 3, and 4 for the test of the condition effect.

		Condition effect					
		True positive	True negative	False positive	False negative	Positive (overlap)	Negative (overlap)
Scenario 1	phoenicsMFA	147 (49%)	2971 (97%)	84 (3%)	153 (51%)	2381 (37%)	4063 (63%)
	phoenicsPCA	182 (61%)	3013 (99%)	42 (1%)	118 (39%)	2307 (36%)	4138 (64%)
	Enrichment	53 (18%)	3055 (100%)	0 (0%)	247 (82%)	296 (5%)	6149 (95%)
	**ktest**	179 (60%)	3055 (100%)	0 (0%)	121 (40%)	2300 (36%)	4145 (64%)
	**globaltest**	0 (0%)	3055 (100%)	0 (0%)	300 (100%)	0 (0%)	6445 (100%)
Scenario 2	phoenicsMFA	112 (37%)	2997 (98%)	58 (2%)	188 (63%)	1735 (27%)	4710 (73%)
	phoenicsPCA	140 (47%)	3026 (99%)	29 (1%)	160 (53%)	1619 (25%)	4826 (75%)
	Enrichment	25 (8%)	3055 (100%)	0 (0%)	275 (92%)	183 (3%)	6262 (97%)
	**ktest**	0 (0%)	3055 (100%)	0 (0%)	300 (100%)	0 (0%)	6445 (100%)
	**globaltest**	0 (0%)	3055 (100%)	0 (0%)	300 (100%)	0 (0%)	6445 (100%)
Scenario 3	phoenicsMFA	21 (7%)	3035 (99%)	20 (1%)	279 (93%)	326 (5%)	6119 (95%)
	phoenicsPCA	40 (13%)	3044 (100%)	11 (0%)	260 (87%)	519 (8%)	5962 (92%)
	Enrichment	0 (0%)	3055 (100%)	0 (0%)	300 (100%)	4 (0%)	6441 (100%)
	**ktest**	0 (0%)	3055 (100%)	0 (0%)	300 (100%)	0 (0%)	6445 (100%)
	**globaltest**	0 (0%)	3055 (100%)	0 (0%)	300 (100%)	0 (0%)	6445 (100%)
Scenario 4	phoenicsMFA	0 (0%)	3043 (100%)	12 (0%)	300 (100%)	22 (0%)	6423 (100%)
	phoenicsPCA	1 (0%)	3052 (100%)	3 (0%)	299 (100%)	27 (0%)	6418 (100%)
	Enrichment	0 (0%)	3055 (100%)	0 (0%)	300 (100%)	0 (0%)	6445 (100%)
	**ktest**	0 (0%)	3055 (100%)	0 (0%)	300 (100%)	0 (0%)	6445 (100%)
	**globaltest**	0 (0%)	3055 (100%)	0 (0%)	300 (100%)	0 (0%)	6445 (100%)

Table S4 SimulatedH1_ConditionTime. Counts and percentages of pathways in each category with semi-synthetic data simulation Scenario 1, 2, 3, and 4 for the test of the time effect.

		Time effect					
		True positive	True negative	False positive	False negative	Positive (overlap)	Negative (overlap)
Scenario 1	phoenicsMFA	0 (0%)	3055 (100%)	0 (0%)	300 (100%)	0 (0%)	6445 (100%)
	phoenicsPCA	179 (60%)	2996 (98%)	59 (2%)	121 (40%)	1990 (31%)	4455 (69%)
	Enrichment	41 (14%)	3055 (100%)	0 (0%)	259 (86%)	217 (3%)	6228 (97%)
	**ktest**	0 (0%)	3055 (100%)	0 (0%)	300 (100%)	3 (0%)	6442 (100%)
	**globaltest**	222 (74%)	2973 (97%)	82 (3%)	78 (26%)	3622 (56%)	2823 (44%)
Scenario 2	phoenicsMFA	0 (0%)	3055 (100%)	0 (0%)	300 (100%)	0 (0%)	6445 (100%)
	phoenicsPCA	168 (56%)	3004 (98%)	51 (2%)	132 (44%)	1781 (28%)	4664 (72%)
	Enrichment	45 (15%)	3055 (100%)	0 (0%)	255 (85%)	247 (4%)	6198 (96%)
	**ktest**	0 (0%)	3055 (100%)	0 (0%)	300 (100%)	0 (0%)	6445 (100%)
	**globaltest**	215 (72%)	2976 (97%)	79 (3%)	85 (28%)	3466 (54%)	2979 (46%)
Scenario 3	phoenicsMFA	0 (0%)	3055 (100%)	0 (0%)	300 (100%)	0 (0%)	6445 (100%)
	phoenicsPCA	139 (46%)	3006 (98%)	49 (2%)	161 (54%)	1327 (21%)	5118 (79%)
	Enrichment	28 (9%)	3055 (100%)	0 (0%)	272 (91%)	162 (3%)	6283 (97%)
	**ktest**	0 (0%)	3055 (100%)	0 (0%)	300 (100%)	0 (0%)	6445 (100%)
	**globaltest**	189 (63%)	2980 (98%)	75 (2%)	111 (37%)	3125 (48%)	3320 (52%)
Scenario 4	phoenicsMFA	0 (0%)	3055 (100%)	0 (0%)	300 (100%)	0 (0%)	6445 (100%)
	phoenicsPCA	68 (23%)	3028 (99%)	27 (1%)	232 (77%)	502 (8%)	5943 (92%)
	Enrichment	7 (2%)	3055 (100%)	0 (0%)	293 (98%)	43 (1%)	6402 (99%)
	**ktest**	0 (0%)	3055 (100%)	0 (0%)	300 (100%)	0 (0%)	6445 (100%)
	**globaltest**	144 (48%)	3000 (98%)	55 (2%)	156 (52%)	2507 (39%)	3938 (61%)

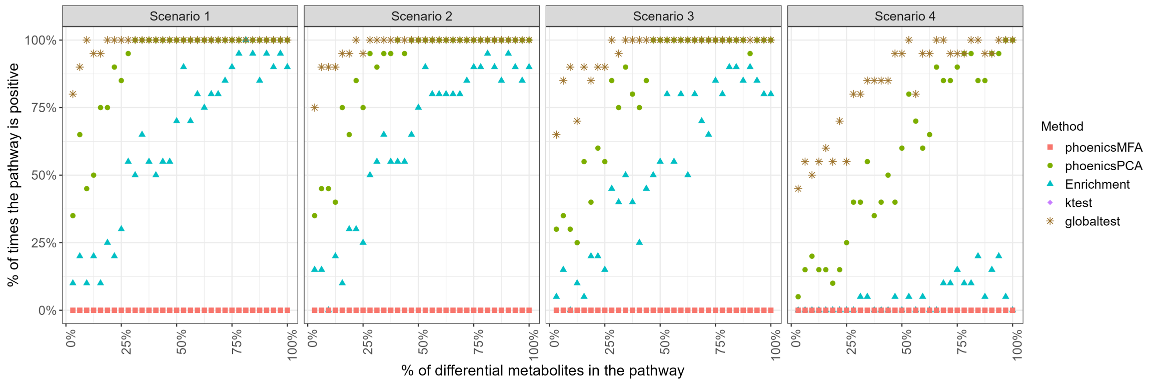

Fig. S3 SimulatedVSize_ConditionTime. Percentage of times the pathway “ABC transporters” is detected positive for the test of the time effect over the 20 simulations with respect to the percentage of differential metabolites in the pathway.

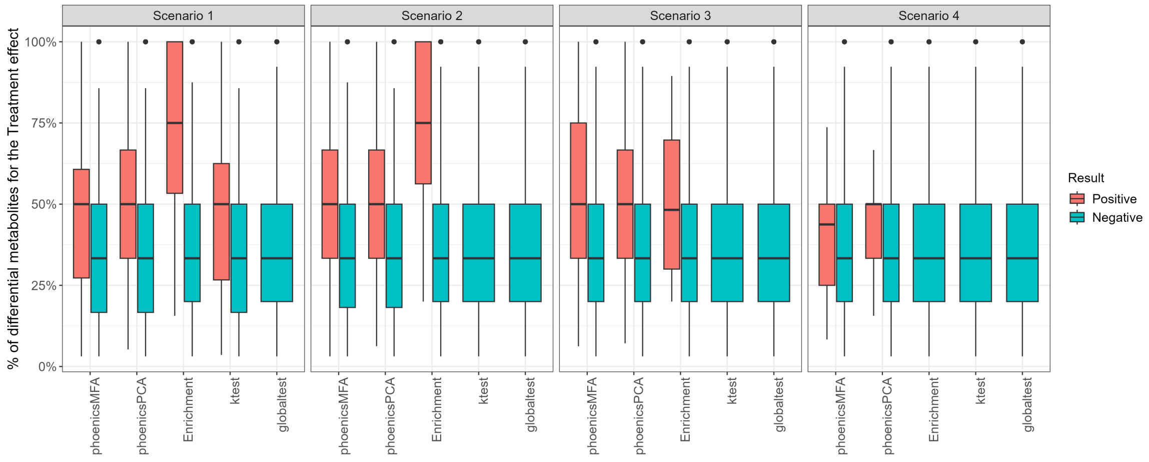

Fig. S4 SimulatedH1_ConditionTime. Percentage of differential metabolites for the condition effect in the overlapping pathways. Some of these pathways have a percentage of differential metabolites equal to 100% because they are included in a differential pathway.

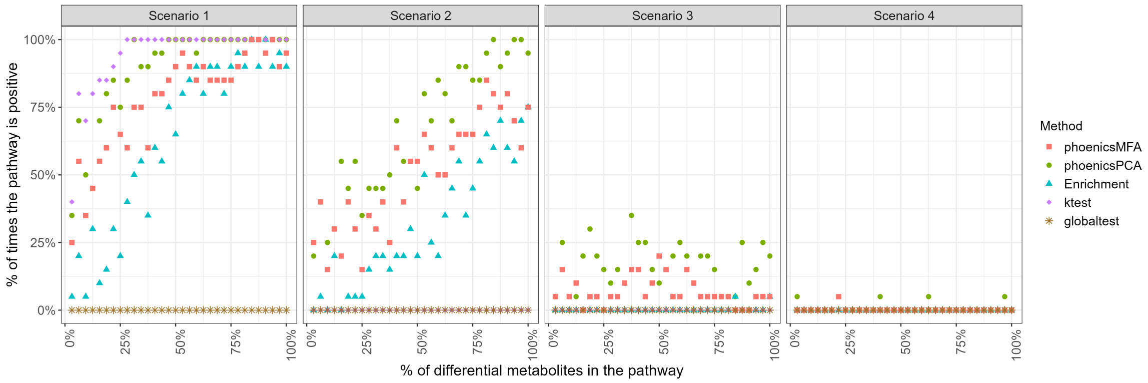

Fig. S5 SimulatedVSize_ConditionTime. Percentage of times the pathway "ABC transporters" is detected positive for the test of the condition effect over the 20 simulations with respect to the percentage of differential metabolites in the pathway.
